# Hepatitis-B-Impfstoffe – Geschichte, Erfolge, Herausforderungen und Perspektiven

**DOI:** 10.1007/s00103-021-03484-w

**Published:** 2022-01-11

**Authors:** Wolfram H. Gerlich

**Affiliations:** Institut für Medizinische Virologie, Nationales Referenzzentrum für Hepatitis-B-Viren und Hepatitis-D-Viren, Justus-Liebig-Universität Gießen, Schubertstr. 81, 35392 Gießen, Deutschland

**Keywords:** Protektive Epitope, HBV, HBsAg, PräS1, Neutralisierende Antikörper, Protective epitopes, HBV, HBsAg, PreS1, Neutralizing antibody

## Abstract

Die ersten Impfversuche gegen das Hepatitis-B-Virus (HBV) erfolgten 1970, noch bevor die Natur des dafür verwendeten „Australia-Antigens“ bekannt war. Bald darauf wurde dieses Antigen als Hüllprotein des HBV erkannt (HBV Surface Antigen, HBsAg), dann aus HBV-haltigem Plasma gereinigt und später gentechnisch in Hefezellen hergestellt. Die hohe Wirksamkeit des HBsAg-Impfstoffs wurde vielfach bewiesen, insbesondere bei Neugeborenen von HBV-infizierten Müttern, die sonst fast immer chronische HBV-Träger werden. Auch bei älteren Kindern und Erwachsenen schützt die Impfung und wird seit 1984 weltweit angewendet, was zu einer ungefähr 10-fachen Abnahme der HBV-Infektionen bei den Geimpften geführt hat.

Es gibt dennoch verschiedene Herausforderungen bei der Hepatitis-B-Impfung. Bei Neugeborenen von hochvirämischen Müttern kann die Impfung versagen. Bei verringerter Immunkompetenz kann die Bildung schützender Antikörper ausbleiben, aber auch bei Risikofaktoren wie höherem Alter, Rauchen oder Übergewicht. Frühe Impfstudien belegten, dass Impfstoffe mit dem HBsAg-Subtyp adw2 auch gegen HBV mit anderen HBsAg-Subtypen schützen, neuere Beobachtungen zeigen aber, dass die Schutzwirkung gegen heterologe Subtypen schwächer ist. Gelegentlich werden auch Escape-Mutationen beobachtet.

Die meisten jetzigen Impfstoffe beruhen auf dem Kenntnisstand vor 40 Jahren und könnten wesentlich verbessert werden. Eine Einbeziehung der bislang fehlenden PräS-Domänen der HBV-Hülle in die Impfstoffe würde die wichtigsten schützenden T‑ und B‑Zellepitope einbringen. Die Expression in Säugerzellkulturen verbessert die native Faltung der neutralisierenden HBsAg-Epitope und die Verwendung von regional vorherrschenden HBsAg-Subtypen würde die Schutzwirkung erhöhen. Optimale Adjuvanzien oder Epitopträger könnten die Immunogenität auch für eine HBV-Immuntherapie steigern.

## Abstract (en)

The first experimental vaccinations against hepatitis B virus (HBV) were performed in 1970, even before the nature of the administered “Australia antigen” was known. Soon, it was realized that this antigen was the envelope protein (HBV surface antigen, HBsAg), and it was purified from HBV-containing human plasma. Later, it was produced in genetically engineered yeast cells. The excellent efficacy of the HBsAg vaccine was confirmed in numerous studies, particularly in newborns from HBV-infected mothers who almost always become chronic HBV carriers without vaccination. But the vaccine is also highly effective in older children and adults and has been applied worldwide since 1984, leading to a circa tenfold decrease of HBV infections in the vaccinated.

Still, there are several challenges with hepatitis B vaccination. In newborns from mothers with very high virus load, the vaccine may fail. Recipients who are immunocompromised, older, smokers, or obese may not produce protective antibodies. Early studies suggested that the vaccine with HBsAg subtype adw2 also protected against infections by other subtypes, but recent observations show that the protection is weaker against heterologous subtypes. Occasionally, escape mutations may develop.

Most current HB vaccines are based on the knowledge of 40 years ago and could be significantly improved. Inclusion of the currently neglected preS domains in the HBV envelope would add the most important protective T‑ and B‑cell epitopes to the vaccines. Expression of the HBsAg in mammalian cell cultures would enhance the folding of neutralizing HBsAg epitopes. Use of the regionally prevalent HBsAg subtypes would increase the protection. Optimal adjuvants and epitope carriers may enhance the immunogenicity to the level necessary for immune therapy of chronic hepatitis B.

Die COVID-19-Pandemie hat der Weltöffentlichkeit die Wichtigkeit von wirksamen, verträglichen und weithin verfügbaren Impfstoffen mit großer Eindringlichkeit deutlich gemacht. Die hier zu erörternde Hepatitis-B-Virus-(HBV-)Infektion, die zur Hepatitis B führen kann, ist seit über 130 Jahren bekannt, jedoch hat es – anders als bei COVID-19 – rund 85 Jahre bis zum ersten Impfversuch gedauert. Die geschichtliche Entwicklung der Kenntnisse über das HBV und den Hepatitis-B-(HB-)Impfstoff wurde vom Autor bereits in Englisch beschrieben [1, 2].

Hier sollen zu Beginn die wichtigsten geschichtlichen Aspekte erläutert werden, die bislang erzielten großen Erfolge der HB-Impfung werden zusammengefasst. Im darauffolgenden Abschnitt werden Probleme und Herausforderungen diskutiert und dann die Perspektiven für Verbesserungen der HB-Impfung auf dem neuesten Stand des Wissens in kurzer Form dargestellt. Zum Verlauf der Infektion und Erkrankung sowie zur Diagnose, Therapie und Prävention sei auf ein Lehrbuch der medizinischen Virologie [3] verwiesen sowie auf die anderen Beiträge in diesem Themenheft.

## Die Geschichte der Hepatitis-B-Impfung

Die Meilensteine in der Entwicklung der HB-Impfung von 1985 bis 2021 sind in Infobox 1 dargestellt.

### Frühe Erkenntnisse zur Hepatitis B

Ausbrüche von epidemischer Gelbsucht wurden schon seit dem Altertum immer wieder berichtet. Ein Ausbruch, der aus heutiger Sicht mit großer Wahrscheinlichkeit vom HBV hervorgerufen wurde, wurde von Lürmann sorgfältig beschrieben [4]. Er entstand unter zahlreichen Werftarbeitern in Bremen, nachdem sie gegen Pocken mittels menschlicher „Lymphe“ geimpft worden waren. Diese „Lymphe“ wurde mutmaßlich aus den durch das Vacciniavirus erzeugten Hautläsionen von bereits Geimpften erhalten. Neben der Verwendung von einem nicht sterilen Instrument an mehreren Personen könnte auch die intrakutane Übertragung dieser vacciniavirushaltigen (und zufällig auch HBV-haltigen!) Körperflüssigkeit von einer Person auf viele weitere Personen zu dem explosionsartigen HB-Ausbruch beigetragen haben. Später folgten noch viele ähnliche Berichte über Gelbsuchtausbrüche nach Impfungen oder Injektionen von Arzneimitteln. Eine umfassende Beschreibung der größten Hepatitisausbrüche weltweit im 20. Jahrhundert und der daraus gewonnenen Erkenntnisse wurde kürzlich publiziert [5]. Epidemiologische Beobachtungen ließen schon lange vermuten, dass es 2 Typen von Hepatitiserregern gab: Typ A, der vorwiegend fäkal/oral durch Hygienemängel im Trinkwasser oder Lebensmitteln übertragen wurde, und Typ B, der sich im Blutserum der Infizierten befand und für die Übertragung die Blutbahn erreichen musste. Daher wurde Typ B auch als Serumhepatitis bezeichnet. Alle damals verfügbaren Methoden, die Erreger nachzuweisen und zu vermehren, versagten.

Die übertragbare Hepatitis wurde in den USA während des 2. Weltkriegs und bis in die 1960er-Jahren als ein derart schwerwiegendes Problem empfunden, dass sogar mehrere Experimente an sogenannten Freiwilligen, z. B. Strafgefangenen, durchgeführt wurden, wodurch die infektiöse Natur der Erkrankung definitiv belegt werden konnte. Eine neue Dimension der damit verbundenen ethischen Problematik wurde jedoch erst 1967 mit den gezielten Infektionsversuchen von Saul Krugman und Mitarbeitern an den Insassen eines Heims für geistig behinderte Kinder (der Willowbrook State School) erreicht [5–7]. Der entscheidende Durchbruch kam jedoch von anderer, unerwarteter Seite.

### Entdeckung des Australia- bzw. Serumhepatitis-Antigens

Der amerikanische Arzt, Genetiker (und spätere Nobelpreisträger) Baruch Blumberg hat die Entdeckungsgeschichte des „Australia-Antigens“ in seiner Autobiografie beschrieben [8]. Er hatte Anfang der 1960er-Jahre nach molekularen Merkmalen der genetischen Empfänglichkeit für bestimmte Krankheiten, speziell Krebs, gesucht und dazu unzählige Serumproben aus aller Welt gesammelt. Die moderne Molekularbiologie mittels Nukleinsäuren war noch nicht verfügbar. Stattdessen verwendete er Antikörper als Sonden für molekulare Unterschiede in Serumproteinen. Er postulierte, dass Personen, die häufig Blutprodukte erhalten hatten, Antikörper gegen bestimmte „polymorphe“ Proteine entwickeln würden, die von Person zu Person, genetisch bedingt, gewisse Unterschiede aufweisen.

Blumbergs Mitarbeiter, Harvey Alter (der kürzlich den Nobelpreis als Mitentdecker des Hepatitis-C-Virus erhielt), fand 1965 tatsächlich ein neues Antigen und zwar zuerst bei einem australischen Ureinwohner, was zur Bezeichnung „Australia-Antigen“ führte. Weitere Untersuchungen schienen die Idee, dass es sich um einen genetischen Marker für bestimmte Krankheiten, z. B. Leukämie oder Trisomie, handeln könnte, zu bestätigen, bis zu der Beobachtung von Blumbergs Assistentin Barbara Werner, dass ihr eigenes Blutplasma, welches sie regelmäßig als Negativkontrolle verwendet hatte, plötzlich positiv war; einige Wochen später erkrankte sie an Hepatitis.

Parallel zu Blumbergs Untersuchungen hatte Alfred Prince vom New York Blood Center auch nach Serummarkern gesucht, allerdings für eine Infektion mit dem Erreger der „Serumhepatitis“, da dessen Übertragung durch Blut- und Plasmaprodukte damals besonders in den USA ein riesiges Problem war. Mit dem gleichen experimentellen Ansatz wie Blumberg fand auch er ein Antigen (von ihm „SH-Antigen“ genannt) im Blutserum von zunächst gesunden Patienten, die Blutprodukte erhalten hatten und später an einer Serumhepatitis erkrankten. Er zeigte zudem, dass das SH-Antigen auch in den Seren von den Kindern vorlag, die 1971 durch den Kinderarzt Saul Krugman gezielt infiziert worden waren [9]. Bald zeigte sich, dass das SH-Antigen mit dem Australia-Antigen (AuAg) identisch war. Die bahnbrechende Arbeit von Prince zeigte, dass man die Erkenntnisse aus Krugmans unethischen Infektionsversuchen auch auf ethisch vertretbarere Weise hätte erhalten können.

### Die Entwicklung der HB-Impfstoffe aus menschlichem Plasma

#### Erster Versuch zum Au/SH-Antigen als Impfstoff.

Blumberg schreibt in seiner Autobiografie [8], dass er bei seinen Studien zur Verbreitung des AuAg und des entsprechenden Antikörpers das Antigen häufig bei Patienten mit Hepatitis, den Antikörper aber nicht bei Lebergesunden gefunden habe. Das habe ihn auf die Idee gebracht, das AuAg aus dem Blutserum von positiven Blutspendern zu reinigen und als Impfstoff gegen Serumhepatitis zu verwenden. Am 08.10.1969 reichte er ein entsprechendes Patent ein, ohne überhaupt eigene Belege hierfür zu haben.

In anderer Form verfolgte diese Idee auch Krugman, mit Finanzierung durch die U. S. Army [10]. Er wies unter Einbeziehung von 39 3‑ bis 10-jährigen Kindern nach, dass der Erreger der Serumhepatitis nach Erhitzen nicht mehr in den inokulierten Kindern infektiös war, aber das Au/SH-Antigen seine Antigeneigenschaften dabei behielt. Damit war der Weg für seinen Impfversuch gegen Hepatitis B, den ersten überhaupt, vorgezeichnet. Krugman verdünnte das Au/SH-antigenhaltige Serum 1:10, erhitzte es für eine Minute auf 100 °C und injizierte eine oder 2 Dosen dieses „Serums“ an 14 Kinder, die allesamt dadurch nicht infiziert wurden. Dann verabreichte er auch das infektiöse Serum an diese Kinder und verfolgte die Laborparameter. Unter Berücksichtigung aller Infektionsparameter erhielt er einen nur partiellen, aber statistisch signifikanten Schutz bei 8 der 14 Kinder (57 %). Damit erschien es plausibel, dass das Au/SH-Antigen ein schützendes Antigen war. Allerdings hatte Prince zuvor zu Recht angemerkt: „Drei Hypothesen können aufgestellt werden: Erstens, SH-Antigen befindet sich auf einem Virus und dieses Virus verursacht einige oder alle Fälle von Serumhepatitis; zweitens, es ist ein virales Antigen, aber nicht Bestandteil des Virus selbst; oder es ist letztlich ein Reaktionsprodukt des infizierten Wirts“ [9]. Krugmans kruder Versuch half nicht, zwischen diesen Alternativen zu entscheiden. Die biophysikalische Charakterisierung des Antigens durch andere Gruppen sprach zunächst nicht für eine Virusnatur. Es befand sich auf rundlichen Teilchen mit ungleichmäßiger Größe von etwa 18–25 nm und enthielt keine Nukleinsäure.

#### Entwicklung des HB-Impfstoffs mit gereinigtem HBV Surface Antigen.

Wissenschaftlich exakter und ethisch vertretbarer waren die Versuche von Robert Purcell und John Gerin an der US-amerikanischen Forschungsbehörde National Institutes of Health (NIH; [11]) und die etwa gleichzeitig anlaufenden Arbeiten des bedeutenden Impfstoffforschers Maurice Hilleman bei der Fa. Merck Sharp & Dohme (MSD; [12]), welche eine Lizenz auf Blumbergs Patent erworben hatte. Beide Gruppen reinigten mit biochemischen Methoden das AuAg aus dem Plasma von positiven Plasmaspendern und inaktivierten die nach der Reinigung eventuell verbliebene Infektiosität mit Formalin und bei MSD später zusätzlich mit Protease und Säure.

Beide Gruppen konnten allerdings auf einem entscheidend besseren Kenntnisstand als Krugman aufbauen. Der britische Pathologe und Virologe David Dane hatte 1970 mittlerweile die nach ihm benannten doppelschaligen 42 nm großen HBV-Partikel in AuAg-positiven Seren entdeckt und das AuAg auf deren Oberfläche mittels „Immunelektronenmikroskopie“ nachgewiesen. Darauf aufbauend konnte die britische Virologin und Elektronenmikroskopikerin June Almeida aus diesen umhüllten Viren das Nukleocapsid mithilfe von Detergenzien freilegen und den dagegen gerichteten Antikörper bei Rekonvaleszenten einer Hepatitis B durch Immunelektronenmikroskopie nachweisen. Diese Entdeckungen führten zur ersten (damals noch nicht endgültig erwiesenen) Identifizierung des HBV und zur heute gültigen Nomenklatur der Hauptantigene des HBV: Hepatitis B Surface Antigen (HBsAg) und HBV Core Antigen (HBcAg). Dass die eigentlichen HBV-Partikel bei den früheren Untersuchungen des AuAg/SH zunächst im Elektronenmikroskop übersehen wurden, lag daran, dass bei typischen HBV-Trägern die Hauptmenge des HBsAg auf den oben erwähnten subviralen 18–25 nm-Partikeln in ca. 3000fachem Überschuss in vergleichsweise riesigen Konzentrationen von 0,01 mg/mL bis zu 1 mg/mL Serum vorliegt. Diese große Menge an HBsAg erlaubte es jedoch erst, Blumbergs Impfstoffkonzept umzusetzen. Die Frage blieb, wie man die Sicherheit und Wirksamkeit eines solchen Impfstoffs beweisen konnte.

Ab 1970 wurden in den USA die Menschenversuche Krugmans zunehmend abgelehnt und ein einfaches Tiermodell oder ein anderes In-vitro-System zum Nachweis von HBV-Infektionen war noch nicht gefunden. Hier half die Beobachtung, dass auch einige Schimpansen, die von der U. S. Army bzw. der Raumfahrtbehörde zu Versuchszwecken gehalten wurden, ohne vorherige gezielte Infektion HBsAg-positiv waren, was für die Empfänglichkeit dieser Tierart für das HBV sprach. In der Tat konnte eine Reihe Untersucher mit diesem Tiermodell den Titer von hochinfektiösen HBV-Seren durch Endverdünnung zu etwa 10^7,5^ 50 % infektiöse Einheiten (ID50) bestimmen. Viele der Tiere überstanden diese experimentelle Infektion ohne größere Erkrankung und entwickelten Antikörper gegen HBsAg (Anti-HBs; [13]). Die Impfung von insgesamt 17 HBsAg- und anti-HBs-negativen Schimpansen mit den beiden neu entwickelten Impfstoffen führte in keinem Fall zu einer HBV-Infektion und zugleich zu einer kräftigen Anti-HBs-Produktion und einem fast kompletten Schutz gegen gezielte Infektion mit 1–3000 ID50 HBV [11, 12]. Damit war der Weg zur Weiterentwicklung und zur Großproduktion des Impfstoffs und zu großen klinischen Studien geebnet.

### Klinische Studien mit HBsAg-Impfstoffen aus Plasma

#### Hämodialyse.

Den ersten Bericht über eine erfolgreiche Erprobung eines HBsAg-Impfstoffs aus Plasma am Menschen publizierte 1976 die Gruppe von Philippe Maupas am Institute Pasteur in Paris [14]. Der Impfstoff entsprach im Wesentlichen den Präparationen von NIH und MSD. Die Erprobung erfolgte an Patienten und Personal von Hämodialyseeinheiten, die in diesen Jahren sehr häufig mit Hepatitis B infiziert waren. Die Ergebnisse bestätigten die Wirksamkeit der Impfung nicht nur beim Personal, sondern auch bei den Patienten, die nach der Impfung innerhalb von 6 Monaten gar nicht mehr infiziert wurden, während die nicht geimpfte Kontrollgruppe zu 43 % HBsAg-positiv wurde. Ein Kritikpunkt an dieser Studie war, dass es keine randomisierte oder verblindete Kontrollgruppe gab, jedoch wurden diese Untersuchungen später entsprechend ergänzt.

#### Männliche Homosexuelle.

Bald darauf, im Jahr 1980, führte der amerikanische Epidemiologe Wolf Szmuness eine vorbildliche placebokontrollierte Doppelblindstudie mit dem MSD-Impfstoff an 1083 homosexuellen Männern in New York durch [15]. Diese von HBV stark betroffene Personengruppe wurde durch den Impfstoff hervorragend geschützt, ohne dass es zu wesentlichen Nebenwirkungen kam. Innerhalb von 18 Monaten wurden in der Placebogruppe 18 % der Probanden HBsAg-positiv, bei der geimpften Gruppe aber nur 1,4 %, was einer Schutzrate von 92,3 % entspricht.

#### Sicherheitsbedenken.

Nach diesem bahnbrechenden Feldversuch schien es keine Hemmnisse für eine umfassende weltweite Einführung des Plasmaimpfstoffs mehr zu geben. Eine kritische Analyse der Sicherheit des MSD- oder ähnlicher Impfstoffe legte jedoch nahe, dass das Vorliegen von HBV-Restinfektiosität durch die Versuche an den wenigen verfügbaren Schimpansen schon aus statistischen Gründen nicht ausgeschlossen werden konnte. Im Ausgangsmaterial auch nur einer einzigen Impfdosis konnten über 100 Mio. infektiöser HBV-Partikel vorliegen. Dazu kam, dass die Impfung schließlich Hunderten Millionen von Empfängern verabreicht werden sollte. Die von NIH und MSD verwendete Inaktivierung mit 1:4000 verdünntem Formalin hatte schon früher bei der inaktivierten Poliovakzine das als Verunreinigung vorliegende DNA-haltige SV40-Tumorvirus nicht ausreichend inaktiviert und zu dessen häufiger Übertragung geführt, was zum Glück wohl folgenlos geblieben ist. Noch bedenklicher war es, dass das Ausgangsmaterial für den MSD-Impfstoff Anfang der 1980er-Jahre zum großen Teil aus den Plasmaspenden von schon HBV-infizierten männlichen Homosexuellen stammte, bei denen sich bald nach der Spende nicht selten eine Aidserkrankung herausstellte. Da das humane Immundefizienzvirus (HIV) erst 1983 identifiziert wurde, konnte eine HIV-Infektiosität des Impfstoffs zunächst nicht ausgeschlossen werden. Auch das Hepatitis-C-Virus (HCV) war damals schon in amerikanischen Plasmaspendern ein größeres Problem als in Deutschland und noch nicht identifiziert und somit auch ein potenzieller Kontaminant.

#### Der Göttinger HB-Impfstoff.

In dieser Situation entschloss sich Reiner Thomssen Ende der 1970er-Jahre an der Universität Göttingen einen eigenen HB-Impfstoff aus dem Plasma deutscher HBsAg-positiver Spender herzustellen. Die HB-Viruslast im Ausgangsplasma wurde durch die ausschließliche Verwendung von gesunden Blutspendern, die den Antikörper gegen das HBV-e-Antigen (Anti-HBe) aufwiesen, wahrscheinlich um den Faktor 1000 oder mehr gesenkt. Die Entfernung der HBV-Partikel wurde mit optimierten Trennmethoden verbessert und der gereinigte HBsAg-Impfstoff wurde mit 4fach stärker konzentriertem Formalin behandelt. Anders als der MSD-Impfstoff wurde der Göttinger Impfstoff nicht mit Proteasen behandelt, was sich später als günstig erwies. Dieser Impfstoff wurde vom Paul-Ehrlich-Institut als Referenzimpfstoff bei der Prüfung von Impfstoffchargen verschiedener Hersteller verwendet und zur Erprobung am Menschen zugelassen. Er wurde bei rund 3000 Probanden ohne jegliche Probleme angewendet und hat in einigen Kollektiven höhere Anti-HBs-Titer als der MSD-Impfstoff induziert [16]. Die Patentansprüche von MSD verhinderten jedoch die weitere Anwendung. Nicht zuletzt aufgrund der Bedenken Thomssens wurde die Sicherheit des MSD-Impfstoffs nach dem ersten großen Feldversuch noch gründlicher überprüft. Die Gefahr der Übertragung einer Aidserkrankung durch Plasmaspenden war durch die Entdeckung des HIV entschärft und die HBV-Infektionssicherheit jedes einzelnen der 3 Inaktivierungsschritte wurde durch zusätzliche Tierversuche mit Schimpansen belegt, sodass eine HBV-Inaktivierung um den überaus reichlichen Faktor von 1:> 10^15^ bewiesen werden konnte [17].

#### Der niederländische HBsAg-Impfstoff.

Die energisch durchgesetzten Patentansprüche von MSD verhinderten zunächst die Produktion der HBsAg-Impfstoffe aus Plasma an anderen Orten. Die Gruppe von Henk Reesink beim niederländischen Blutspendedienst in Amsterdam umging das Patent dadurch, dass sie die alte Idee von Krugman aufgriff. Sie verwendete *weitgehend ungereinigtes *HBsAg-haltiges Plasma als Impfstoff, nachdem das etwas angereicherte HBsAg zunächst 10 h auf 65 °C und dann 90 sec auf 100 °C erhitzt worden war. Obwohl eine Dosis dieses Impfstoffs nur 3 µg HBsAg enthielt anstatt 20–40 µg wie bei den vorher erwähnten Impfstoffen, reichte die Immunogenität, um im Jahr 1983 die Wirksamkeit in einem Kollektiv von homosexuellen Männern zu beweisen [18].

#### Impfung von Neugeborenen HBV-infizierter Mütter.

Die erfolgreiche Impfung von erwachsenen Personen mit hohem Risiko für eine HBV-Infektion bzw. eine schwere HBV-Erkrankung war ein großer Erfolg, der jedoch in seiner Bedeutung noch deutlich hinter der überraschend guten Wirksamkeit der Impfung bei Neugeborenen von HBV-infizierten Müttern einzustufen ist, denn die perinatale Infektion wird bei 70–90 % zur chronischen HBV-Infektion, die das eigentliche Erregerreservoir aufrechterhält und im späteren Leben häufig zu Leberzirrhose, Leberversagen und hepatozellulärem Karzinom (engl. HCC) führt.

Bevor die aktive Impfung in großem Stil eingesetzt wurde, wurde im Jahr 1981 von den amerikanischen Epidemiologen Palmer Beasley, Cladd Stevens und Kollegen die passive Immunisierung dieser Neugeborenen innerhalb von 12–24 h nach der Geburt mit anti-HBs-haltigem menschlichen Immunglobulin (HBIG) mit überraschendem Erfolg angewendet. Überraschend war der Erfolg insofern, als der Fetus schon monatelang der massiven Virämie der Mutter ausgesetzt war und spätestens bei der Geburt wesentliche Mengen mütterlichen Bluts in das Neugeborene übergetreten waren. Ein Schwachpunkt dieser Immunisierung waren gelegentliche Durchbrüche des Virus. Zudem verschwand der Schutz innerhalb weniger Monate und die Kinder blieben weiter im infektiösen Umfeld ihrer Familie. Die beteiligten Ärzte erkannten, dass die passive durch eine aktive Immunisierung ergänzt werden musste, um eine hohe Schutzrate zu erhalten [19].

Pionier bei der aktiven Impfung von Kindern unter 2 Jahren war wiederum Philippe Maupas mit einer Studie im Senegal, wo es eine sehr hohe HBV-Prävalenz gab [20]. Die überzeugendsten Impfkampagnen für Neugeborene mit der weitgehenden Verhinderung der perinatalen Übertragung durch hochvirämische Mütter begannen jedoch 1984 in Taiwan und den USA unter Beteiligung von Palmer Beasley und Cladd Stevens [19]. Zunächst wurden die Plasmaimpfstoffe verwendet, sowohl von MSD als auch aus den Niederlanden. Bald wurden jedoch diese Impfstoffe durch den sogenannten rekombinanten Impfstoff ersetzt.

## Entwicklung der gentechnisch hergestellten Hepatitis-B-Impfstoffe

### Aufklärung der HBsAg-Struktur.

Bald nach der Entdeckung des HBsAg begann dessen biochemische Charakterisierung. Gereinigte HBsAg-Partikel mit 18–25 nm Durchmesser zeigten in der SDS-Gelelektrophorese 2 gut erkennbare Proteinbanden mit 24.000 und 27.000 Molekulargewicht, was von mehreren Autoren übereinstimmend berichtet wurde. Daneben zeigte sich eine Reihe weiterer größerer Proteine, über die jedoch keine Einigkeit bestand. Wurde das HBsAg vor der Reinigung mit Proteasen behandelt, wie z. B. der MSD-Impfstoff, verschwanden diese Proteine, sodass viele Autoren sie für verunreinigende Serumproteine hielten. Schließlich konnten 1977 Darrell Peterson und Girish Vyas von der Universität in San Francisco eine teilweise Peptidsequenz der beiden Proteine aufklären, wobei sich zeigte, dass sie die gleiche Sequenz hatten und die größere Form lediglich eine zusätzliche Glycosidseitenkette enthielt [21]; oft werden diese beiden Proteine als P24 und GP27 entsprechend ihrem Molgewicht in Kilodalton bezeichnet, wobei G für Glyco steht.

Ein denkbarer Weg zu einem synthetischen Impfstoff war die chemische Herstellung von HBsAg-Teilpeptiden, die eventuell die wesentlichen HBsAg-Epitope enthielten. Entsprechende Versuche wurden vielfach angestellt, jedoch reagierten diese Peptide kaum oder gar nicht mit den Anti-HBs-Antikörpern, die von Hepatitis-B-Rekonvaleszenten oder von mit Plasmaimpfstoff geimpften Personen kamen. Das native HBsAg weist offensichtlich nur konformationelle B‑Zellepitope auf. Vyas hatte zuvor schon gezeigt, dass die natürliche HBs-Antigenreaktivität von der komplexen Faltung der Proteinkette und von stabilen Disulfidvernetzungen im HBsAg-Protein abhing [22].

### Identifizierung der HBV-DNA.

Eine Vermehrung der HBV-Partikel in Zellkulturen oder verfügbaren Versuchstieren außer Schimpansen war lange Zeit trotz intensivster Bemühungen nicht gelungen. Neue Wege eröffnete die zunächst biochemische Identifizierung der HBV-DNA in den HBV-Partikeln aus infizierten Patienten oder aus asymptomatischen Spendern. Bei der Überprüfung eines nicht reproduzierbaren Berichts über eine reverse Transkriptase in HBV-Proben gelang es William S. Robinson von der Stanford University 1973 in den teilgereinigten Viruspräparationen eine ungewöhnliche „endogene“ DNA-Polymeraseaktivität nachzuweisen und schließlich im Elektronenmikroskop die nur 3200 Basen lange ringförmige HBV-DNA selbst [23].

### Klonierung und Sequenzierung der HBV-DNA.

Es dauerte noch weitere 5 Jahre, bis die mittlerweile erfundenen Methoden der DNA-Klonierung auf das HBV angewendet werden konnten, da auf der berühmten Konferenz von Asilomar, USA, aus prinzipiellen Sicherheitsbedenken ein Moratorium für gentechnische Experimente mit pathogenen Organismen in den USA verabschiedet wurde. Schließlich hatten 3 Pioniere der modernen Molekularbiologie, Pierre Tiollais am Institute Pasteur in Paris, William Rutter in San Francisco und Kenneth Murray in Edinburgh, Hochsicherheitslabors für ihre Arbeiten zur Klonierung der HBV-DNA eingerichtet und 1979 fast gleichzeitig ihre Erfolge publiziert. Die Klonierung ermöglichte unmittelbar die Herstellung großer Mengen der HBV-DNA, die Bestimmung ihrer Sequenz und die Identifizierung der HBV-Gene [24–26]. Wenn es noch eines Beweises bedurft hätte, dass die von David Dane entdeckten Partikel wirklich das HBV darstellten, wurde er 1982 von den deutschen Forschern Hans Will, Friedrich Deinhardt und Heinz Schaller erbracht. Sie injizierten die klonierte HBV-DNA in die Lebern zweier Schimpansen und erhielten in beiden Tieren alle Merkmale einer HBV-Infektion [27]. Die direkte Vermehrung von infektiösem HBV in suszeptiblen Leberzellkulturen gelang dagegen erst Jahrzehnte später.

### Expression des HBsAg in transfizierten Zellkulturen.

Rascher gelang die Produktion von HBsAg in Wirtszellen aller Art ab 1980. Die erste Mitteilung einer Expression des HBsAg in Bakterien, die mit dem Gen für HBsAg transfiziert waren (als Teil der Patentanmeldung der Fa. Biogen basierend auf der Arbeit von K. Murray), war jedoch vorschnell, da HBsAg von Bakterienzellen nicht toleriert wird. Die Expression des HBsAg in Form des P24/GP27 nach Transfektion eines Vektors, der das Gen für P24 enthielt, war dagegen in verschiedenen nichthepatischen Säugerzellkulturen unproblematisch und führte zur Sekretion von nativen HBsAg-Partikeln mit dem natürlichen Verhältnis von P24 zu GP27 und mit allen konformationellen Epitopen [28]. Die Konzentration an HBsAg im Überstand dieser Zellkulturen war mit maximal 0,25 µg/mL leider eher niedrig, verglichen mit den rund 100- bis 1000fach höheren HBsAg-Mengen im Plasma von HBV-Trägern mit hoher HBs-Antigenämie. Dies mag der wesentliche Grund dafür sein, dass es bislang nur wenige Beispiele für einen HB-Impfstoff aus Säugerzellkulturen gibt.

### HBsAg-Produktion in transformierten Hefezellen.

Bald stiegen die 2 großen Pharmakonzerne MSD und GlaxoSmithKline (GSK) auf die Expression des HBsAg in Hefezellen um, wobei die Hefekultur weniger anspruchsvoll und zusätzlich die Ausbeute viel höher ist. Es stellte sich allerdings heraus, dass die Hefezellen das HBsAg nicht sezernierten, das P24 nicht glykosyliert war und nach der Extraktion die schützenden konformationellen Epitope des HBsAg fehlten, die sich bei einem Teil der P24-Moleküle erst nach einem wenig definierten Reifungsprozess bildeten. Trotzdem waren die Hefeimpfstoffe in Schimpansen, die damit geimpft worden waren, sehr wirksam, sodass diese Tiere vollständig gegen experimentelle Infektionen mit HBV geschützt waren. Bald danach wurden diese Impfstoffe in großen Feldversuchen mit Neugeborenen von hochvirämischen Müttern eingesetzt. Cladd Stevens fand bei der passiv/aktiven Immunisierung eine Gleichwertigkeit des Hefeimpfstoffs mit dem Plasmaimpfstoff und nur bei 4,8 % der immunisierten Neugeboren (statt 70–90 % bei den Ungeimpften) später eine chronische HBV-Infektion [19, 29]. Besonders bemerkenswert war der Feldversuch von Y. Poovorawan in Thailand, wobei keine zusätzliche passive Immunisierung verwendet wurde. Nur 2 von 55 geimpften Neugeborenen entwickelten eine chronische HBV-Infektion statt der bei den Ungeimpften zu erwartenden 32–50 HBV-Infektionen [30].

### Vielfalt der verfügbaren Hepatitis-B-Impfstoffe.

Eine Übersicht über Impfstoffe, die in der Praxis weltweit oder regional verwendet werden, ist in Referenz [31] gegeben. Die meisten Impfstoffe sind gentechnisch in Hefezellen unter Verwendung desjenigen HBsAg-P24-Gens erzeugt, das von Rutters Gruppe identifiziert wurde. Auch die Marktführer GSK und MSD verwenden mit ihren Impfstoffen Engerix-B® bzw. Recombivax HB® diese Methodik. Einen Überblick über die wichtigsten älteren und neueren HB-Impfstoffe gibt Tab. 1.

**Table 1:** 

Jahr	Name, Hersteller, Verfügbarkeit	Art des Antigens, Adjuvans, HBsAg-Subtyp	Lit.
*HBsAg aus humanem Plasma mit Aluminiumverbindung, unklarer Subtyp*			
1971	Krugmans „Vakzine“ (exp.)	MS2 Serum, 1 min. auf 100 °C erhitzt	[10]
1976	Hevac B, Institut Pasteur (n.v.)	Gereinigtes HBsAg aus HBeAg-neg. Plasma	[14, 20]
1980	Heptavax®, MSD (n.v.)	Gereinigtes HBsAg aus HBeAg-pos. Plasma	[15, 17]
1982	Göttinger Vakzine (n.v.)	Gereinigtes HBsAg aus HBeAg-neg. Plasma	[16]
1983	Niederländische Vakzine (n.v.)	HBV-Trägerplasma, HBeAg+, 2fach erhitzt	[18]
*S‑HBsAg-P24 aus transformierter Hefe, verschiedene Adjuvanzien, Subtyp adw2*			
1982	Recombivax HB®, MSD (z.)	S‑HBsAg-Protein, Al-Verbindung	[2, 31]
1983	Engerix-B®, GSK (z.)	S‑HBsAg-Protein, Al-Verbindung	[2, 31]
2008	Fendrix®, GSK (z.)	S‑HBsAg-Protein, MP-Lipid A (TLR4-Agonist)	[32]
2018	Heplisav-B®, Dynavax (z.)	1 HBsAg-Protein, CpG (TLR9-Agonist)	[33, 34]
*HBsAg aus Säugerzellkultur mit teilglykosylierten Proteinen und Al-Verbindung*			
1980	GenHevac B®, Inst. Pasteur (n.v.)	2 HBsAg-Proteine (S, M) *ayw*	[28]
1996	Sci-B-Vac®, VBI Vaccines (z.)^a^	3 HBsAg-Proteine (S, M, L) *adw*	[35, 36]
2001	Hepagene®, Medeva (n.v.)	3 HBsAg-Proteine (S, M, L) *adw *und* ayw*	[2, 37]
*Neue Konzepte*			
2016	BM32®, Viravaxx (exp.)	PräS-Graspollenallergen Fusionsprotein	[38, 39]

*Al.* Aluminium, *exp.* experimentell, *HBeAg* Hepatitis B e Antigen, *HBsAg* Hepatitis B Surface Antigen, *L* Large, *M* Middle, *neg.* negativ, *n.v.* nicht mehr verfügbar, *pos.* positiv, *S* Small, *TLR* Toll-like-Rezeptor, *z.* zugelassen

^a^ Zugelassen in Israel und den USA, Zulassung durch die Europäische Arzneimittelagentur (EMA) steht noch aus

## Erfolge der Hepatitis-B-Impfung

Eine Auflistung aller Erfolge liegt außerhalb der Möglichkeiten dieses kurzen Beitrags. Seit dem Jahr 1984 wurde die Hepatitis-B-Impfung für Kinder und zunehmend auch für Neugeborene in immer mehr Ländern eingeführt. Laut Fortschrittsbericht der Weltgesundheitsorganisation (WHO) zu HIV, Virushepatitis und anderen sexuell übertragbaren Infektionen (STI) von 2021 haben fast alle Länder diese Impfprogramme eingeführt (außer Island und Finnland – wegen kaum vorhandener HBV-Prävalenz; [40]). Bei sachgerechter Durchführung gibt es keine wesentlichen Zweifel an der Wirksamkeit dieser Maßnahmen.

Die Impfquote bei Kindern mit allen 3 empfohlenen Dosen soll im Jahr 2020 laut WHO 90 % erreicht haben, wobei die HB-Impfung Teil einer penta- oder hexavalenten Impfung gegen eine Reihe von pädiatrisch relevanten Erregern ist. Zu gering ist mit ca. 50 % noch die Verabreichung monovalenter HB-Impfungen bei Neugeborenen von HBsAg-positiven Müttern oder bei unbekanntem maternalen HBsAg-Status direkt nach der Geburt („birth dose“). Die weltweit geschätzte Prävalenz von HBsAg-positiven HBV-Infektionen bei den unter 5‑jährigen Kindern liegt zurzeit (2021) bei 0,94 % [40], während sie in den Jahren 1980–2000 vor der breiten Einführung der Impfung auf 4,7 % geschätzt wurde [41]. Regional kann die Erfolgsrate je nach Ausgangslage und Einführung der Impfung jedoch deutlich höher sein.

In einem Überblick von 2010 berichtet Din-Shinn Chen, einer der Pioniere der HB-Impfung in Taiwan, dass in 13 von 19 Ländern mit entsprechenden Studien die HBsAg-Prävalenz nach Einführung der Impfung um mehr als 90 % bis zu 100 % abnahm [42]. In Taiwan wurde die allgemeine Impfung der Neugeborenen 1984 eingeführt. 30 Jahre später waren die ungeimpften über 30-Jährigen zu 6,7 % HBsAg-positiv, die etwas jüngeren Geimpften nur noch zu 0,5 % [43]. Darüber hinaus zeigte sich schon bei den 6‑ bis 19-jährigen Geimpften gegenüber den ungeimpften Gleichaltrigen eine 3fach niedrigere Inzidenz des HBV-assoziierten hepatozellulären Karzinoms [44]. Damit kann die Hepatitis-B-Impfung als erste Impfung gegen eine menschliche Krebserkrankung bezeichnet werden. Die großen Erfolge der Impfung bei erwachsenen Personen mit hohem beruflichen oder medizinischem Expositionsrisiko wurden bereits oben kurz erwähnt.

Jetzt hat die WHO das durchaus realistische Ziel vorgegeben, die Inzidenz neuer HBV-Infektionen bis 2030 um 90 % zu reduzieren [40]. Zu dessen Erreichung ist nicht nur eine konsequente Impfkampagne nötig, sondern es sind auch verbesserte Impfstoffe wünschenswert.

## Herausforderungen

Es gibt eine Reihe von Situationen, bei denen die Impfung ganz oder teilweise versagt, die hier kurz diskutiert werden sollen.

### Impfdurchbrüche bei der perinatalen HBV-Übertragung.

Die frühen Studien von 1981–1993 zur passiv/aktiven Immunisierung von Neugeborenen von HBsAg- und HBeAg-positiven Müttern mit sehr hoher Viruslast in den USA zeigten innerhalb eines Jahres nach der Geburt bei 7,3–14,4 % der Kinder eine chronische HBV-Infektion trotz sehr rascher HBIG-Gabe und vollständiger aktiver HB-Impfung. Dabei zeigte sich, dass der gentechnisch hergestellte MSD-Impfstoff trotz 4fach geringerer Dosierung eher besser wirkte als der MSD-Plasmaimpfstoff. Es zeigte sich außerdem, dass Durchbrüche ausschließlich bei Müttern mit einer HBV-Last > 1 Mio. HBV-DNA-Molekülen/mL vorkamen [19].

Die große Häufigkeit von Durchbrüchen bei hoher maternaler Virämie um 100 Mio. HBV-DNA-Molekülen/mL wurde in einer Studie von 2013 in den USA mit 18 % (15/88) bestätigt [45], während eine andere Studie in Thailand bei etwa gleicher maternaler Viruslast nur 2 % (3/147) fand [46]. Anzumerken ist, dass die Studie in den USA den Kindern nur die üblichen 3 Dosen des aktiven Impfstoffs verabreichte, die Studie in Thailand jedoch 5 Dosen gab. Eine Studie in Vietnam von 2010 fand andererseits sogar 33,8 % (23/68) Durchbrüche bei Kindern von HBeAg-positiven Müttern, wobei allerdings nur die aktive Impfung direkt nach der Geburt, aber kein HBIG und später nur 2 weitere Dosen gegeben wurden [47].

Bei zwar heterogener Datenlage ist die unvollständige Schutzwirkung der aktiv/passiven Immunisierung von Neugeborenen nicht wegzuleugnen. Daher hat die WHO kürzlich empfohlen, bei HBsAg-positiven Schwangeren die Viruslast oder ersatzweise das HBeAg zu bestimmen und bei hoher Viruslast oder positivem HBeAg die Schwangere ab der 28. Woche mit einem antiviralen Mittel gegen HBV, vorzugsweise mit Tenofovir zur Senkung der Viruslast zu behandeln [48]. Wäre die aktive Immunisierung noch zuverlässiger wirksam, könnte in Ländern mit eingeschränkter Infrastruktur der Schutz der Neugeborenen viel einfacher erreicht werden und die Therapie während der Schwangerschaft mit einem Nucleotidanalogon wie Tenofovir unterbleiben.

### Nützlichkeit der zusätzlichen passiven Immunisierung bei den gefährdeten Neugeborenen.

Es mag auf den ersten Blick dringlich scheinen, das von der Mutter bei der Geburt auf das Kind übertragene HBV so rasch wie möglich zu neutralisieren, jedoch sprechen die Erfolge der Impfstudien ohne zusätzliches HBIG (z. B. in Thailand) dagegen, dass die Leber in den ersten Wochen voll empfänglich für die HBV-Infektion ist. Es ist bekannt, dass die fetale Leber erst nach der Geburt innerhalb von 4 Wochen in die adulte Form umgebaut wird. Eine experimentelle Überprüfung dieser Hypothese wäre wünschenswert.

Alle internationalen Leitlinien und auch die neue S3-Leitlinie der Deutschen Gesellschaft für Gastroenterologie, Verdauungs- und Stoffwechselkrankheiten (DGVS) zur Prophylaxe, Diagnostik und Therapie der Hepatitis-B-Virusinfektion ([49], Empfehlung 5.9.1) sowie der Ständigen Impfkommission (STIKO; [50]) empfehlen eine HBIG-Gabe zusätzlich zur aktiven Impfung bei Neugeborenen von HBsAg-positiven Müttern. Erwiesen ist die Nützlichkeit der HBIG-Gabe jedoch nicht. Für hoch entwickelte Länder mag dies eine Marginalie sein, aber in Ländern mit eingeschränkter Infrastruktur ist das Erfordernis einer durchgängig verfügbaren Kühlkette nur für die sofortige HBIG-Gabe nach der Geburt ein wesentliches Hindernis, während der aktive Impfstoff sehr wärmestabil ist.

### Unzureichende Immunogenität bei älteren oder gesundheitlich beeinträchtigten Personen.

Schon bei den ersten Erprobungen in Erwachsenen zeigte sich, dass mit zunehmendem Alter und mit jeder immunologisch bedeutsamen Beeinträchtigung der Gesundheit die Anti-HBs-Serokonversionsrate immer schlechter wird. Gerade bei den in der Indikationsliste der S3-Leitlinie ([49], Tab. 15, S. 747) genannten Personen ist eine Überprüfung des Anti-HBs-Titers empfohlen, wobei oft ein unzureichender Titer unter 100 IU/L festgestellt werden wird. Für bestimmte Patientengruppen, z. B. Dialysepatienten, wird eine höhere HBsAg-Dosis empfohlen. Bei anderen Gruppen werden zusätzliche Impfdosen empfohlen.

Eine im Grunde unbeantwortete Frage ist, nach welchen objektiven Kriterien bei Wiederholungs- und Auffrischimpfungen die Zahl der Dosen und die Abstände zwischen deren Verabreichung bestimmt werden. Während erster und zweiter Impfung werden übereinstimmend 4–6 Wochen empfohlen, danach 6–12 Monate. Gerade die dritte oder gar vierte Impfung wird oft unterlassen, was zu einem unvollständigen Schutz führt. Ob überhaupt eine Testung auf Anti-HBs und gegebenenfalls eine Auffrischungsimpfung nötig sind, wird international kontrovers diskutiert. Die deutschen Leitlinien beschränken diese Maßnahme auf Personen mit erhöhtem Risiko für eine HBV-Infektion oder für deren schwereren Verlauf.

### Ungleichmäßiger Schutz gegen heterologe HBV-Genotypen.

Die vom HBV genetisch festgelegte immunologische Heterogenität des HBsAg wurde schon 1970 anhand der „allelischen“ Subtypdeterminanten HBsAg *d* oder *y* und *w* oder *r* erkannt. Es war beruhigend, dass alle HBsAg-Proben auch eine gemeinsame Determinante *a* enthielten. Später wurden noch die Subtypdeterminanten *w1–4* unterschieden, welche neuerdings korrekter als *a1–a4* bezeichnet werden sollten [51]. Durch systematische DNA-Sequenzierung von HBV-DNA-Proben weitgestreuter geografischer, ethnischer und medizinischer Herkunft wurden HBV-Genotypen und HBV-Subgenotypen identifiziert, deren Bedeutung für die HB-Impfung bislang noch nicht genügend beachtet wird [51, 52].

Eine frühe große Feldstudie in Hämodialysestationen zeigte, dass der HBsAg-Subtyp *ad* bzw. HBV-Subgenotyp A2 des MSD-Plasmaimpfstoffs auch gegen einen HBV-Stamm mit HBsAg/ay zuverlässig schützte [53]. Danach wurde die universelle Schutzwirkung eines Impfstoffs mit einem HBsAg-Subtyp als gegeben erachtet. Überraschend zeigte sich 2010 bei geimpften, dennoch frisch infizierten Erwachsenen ein HBsAg-Subtyp- bzw. ein HBV-Subgenotypeffekt: Bei einigen frisch infizierten US-amerikanischen Blutspendern mit Impfung zeigte sich gehäuft eine HBV-Infektion mit unterschiedlichen Non-A2-Subgenotypen, während bei den nicht geimpften frisch infizierten Spendern meist der in den USA vorherrschende HBV-Subgenotyp A2 vorlag, der auch für den MSD-Impfstoff verwendet wurde. Man beachte, dass die meisten dieser geimpften und dann infizierten Spender einen nachweisbaren Anti-HBs-Titer hatten, allerdings unter 100 IU/L, was auch die Relevanz einer quantitativen Anti-HBs-Bestimmung belegt [54].

HBsAg-Subtypeffekte könnten eventuell auch die unterschiedlichen Schutzraten der Impfstoffe bei der perinatalen Übertragung erklären. Bei den Impfungen zur Verhinderung perinataler Übertragungen war die Immunisierung mit dem HBsAg/ad im Impfstoff Recombivax HB® gegen HBV mit HBsAg-Subtyp *ad* doppelt so wirksam wie gegen HBV mit HBsAg/ay [19]. In Taiwan wurde nach Einführung der universellen Hepatitis-B-Impfung eine Verschiebung der Prävalenz der HBV-Genotypen nach perinataler Übertragung von B nach C beobachtet, wobei der Genotyp B meistens den gleichen HBsAg-Subtyp *adw2* hat wie der aktive Impfstoff, Genotyp C dagegen *adr *[55]. Auch in China hat sich die Prävalenz der HBV-Genotypen und HBsAg-Subtypen nach Einführung der Impfung verschoben [56]. Einige der HBsAg-Impfstoffe verwenden nicht den Subtyp *adw2* wie MSD und GSK, sondern die regional vorherrschenden Subtypen, wie z. B. in Korea oder Japan HBsAg/*adr* [31]. In Japan wurden bei einer Studie weniger Durchbrüche mit HBsAg-Persistenz (1/158) als in den USA beobachtet [57].

### Okkulte HBV-Infektionen und Escape-Mutationen.

Der erste Bericht einer Escape-Mutation nach passiver und aktiver Impfung kam 1990 aus Italien, wo ein Kind einer HBsAg- und HBeAg-positiven Mutter nach 11 Monaten gleichzeitig HBsAg- und anti-HBs-positiv war, wobei das Anti-HBs später verschwand, das HBsAg jedoch blieb. Das beteiligte Virus hatte eine auffällige Mutation im HBsAg von Glycin (G) nach Arginin (R) in Position 145 (G145R), die offensichtlich die Wirkung der Immunisierung wesentlich verminderte. Diese Mutation wurde noch öfter beobachtet, jedoch spielen diese oder andere Escape-Mutationen noch keine größere Rolle und sollen hier nicht vertieft erörtert werden.

Häufiger werden Mutationen des HBsAg bei reaktivierten okkulten HBV-Infektionen unter Immunsuppressionen, speziell der B‑Lymphozyten nachgewiesen [58]. Bemerkenswert ist ein Fall von Reaktivierung bei einem Lymphompatienten, der Jahre früher gegen HBV geimpft worden war, dann aber in einem endemischen Gebiet inapparent mit HBV infiziert wurde. Die HBV-Infektion wurde okkult und blieb es, bis wegen des Lymphoms eine Depletion der B‑Lymphozyten erforderlich wurde. Im Verlauf entwickelte sich eine massive HB-Virämie mit einer Escape-Mutante, die sich vom HBV-Genotyp D4 ableitete, der bereits 7 Austausche in der HBs-Antigenschleife im Vergleich zum Genotyp A2 im Impfstoff aufweist. Die dann schließlich reaktivierte Variante hatte noch 5 weitere Mutationen [59]. Solche zwar seltenen, dann aber besonders anti-HBs-resistenten Varianten sind ein Grund, stärker und/oder breiter wirksame Impfstoffe zu entwickeln.

Reaktivierungen einer okkulten HBV-Infektion sind insgesamt selten, die okkulte HBV-Infektion selbst ist dagegen sehr häufig, auch nach erfolgreicher Impfung. Poovorawan fand 20 Jahre nach seinem erfolgreichen Schutzversuch bei seinen Probanden zwar keine einzige neue chronische HBV-Infektion, aber 22,8 % hatten Anti-HBc, genauso häufig wie die ungeimpfte Kontrollgruppe [60]. Diese Form der okkulten HBV-Infektion ist an sich unbedenklich, aber das Reaktivierungsrisiko (s. oben) lässt es wünschenswert erscheinen, dass wirksamere HB-Impfungen auch diesen Verlauf möglichst unterbinden, was 2013 auch die STIKO mit folgenden Sätzen beschrieb: „Ziel der Impfung gegen Hepatitis B ist die Verhinderung der akuten klinischen Hepatitis und von chronischer Infektion mit dem Hepatitis-B-Virus (HBV). Darüber hinaus ist eine Verhinderung sämtlicher Infektionsformen (einschließlich okkulter Infektionen) erstrebenswert“ [61].

### Fehlen der PräS-Domänen in den meisten Impfstoffen.

Eine aus heutiger Sicht seltsam anmutende Eigenheit der heute vorwiegend verwendeten Impfstoffe ist es, dass sie nicht diejenige Komponente der HBV-Hülle enthalten, die für den Eintritt des HBV in die Leberzelle unersetzlich ist. Es ist, als ob man einen Impfstoff gegen SARS-CoV‑2 ohne Spike-Protein verwenden würde oder einen Influenzaimpfstoff ohne Hämagglutinin. Die 2 zusätzlichen HBsAg-Proteine zum kleinen („small“) S‑HBs-Protein P24/GP27, das große („large“) L‑HBs-Protein P39/GP42 mit der PräS1-Domäne und das mittlere („middle“) M‑HBs-Protein GP33/GP36 mit der PräS2-Domäne wurden erst 1984 nach der Erprobung und Zulassung der ersten HB-Impfstoffe nachgewiesen [62]. Die schwierige Entdeckung der M- und L‑HBs-Proteine, ihre Funktionen und ihre Immunogenität sind in Referenz [2] genauer beschrieben. Beide PräS-Domänen enthalten neutralisierende Epitope, wobei die Wirkung der PräS1-Antikörper gegen die HBV-Anheftungssequenzen im Bereich der Aminosäuren 2–48 durch Blockade der Bindung an den HBV-Rezeptor „NTCP“, einem hepatischen Gallensäuretransporter, komplett aufgeklärt ist, während die Wirkungsweise von PräS2-Antikörpern unklar bleibt.

### Geringe Beachtung der präS-haltigen Impfstoffe.

Kurzzeitig gab es den präS2-haltigen HB-Impfstoff GenHevac B® vom Institute Pasteur aus Säugerzellkulturen, der aber keine erkennbaren Vorteile hatte. Dies galt auch für einen in Hefezellen produzierten experimentellen HB-Impfstoff der Fa. GSK, der neben dem SHBs-Protein auch PräS1- und PräS2-Epitope enthielt. 2 Impfstoffe aus Säugerzellkulturen (Hepagene® bzw. Hepacare von Medeva und Bio-Hep-B3 bzw. Sci-B-Vac® aus Israel), die alle 3 HBs-Proteine (S, M, L) mit allen 3 Hüllproteindomänen in ausgewogenem Verhältnis enthielten, erzeugten dagegen in mehreren Studien eine deutlich bessere Serokonversionsrate und höhere Anti-HBs-Titer als die konventionellen Impfstoffe mit P24 aus Hefe [2, 37]. Ein Vorteil der präS-haltigen Impfstoffe ist die größere Zahl an T‑Zellepitopen als bei reinen S‑HBs-Impfstoffen. Schon bald nach der Entdeckung der PräS-Proteine zeigten Milich et al. in bestimmten Mausstämmen, dass eine fehlende Anti-HBs-Bildung gegen S‑HBs durch zusätzliche PräS-Komponenten über eine verbesserte T‑Zellhilfe behoben werden konnte [63].

Die beiden präS1-haltigen Impfstoffe nahmen eine unterschiedliche Entwicklung. Die Fa. Medeva wurde von der Fa. Powderject übernommen, die das Projekt kurzerhand beendete. Der israelische Impfstoff wurde in Israel erfolgreich eingeführt, aber blieb mit einigen wenigen Ausnahmen international lange Zeit weitgehend unbeachtet.

### Unbefriedigende Bestimmung HBV-neutralisierender Antikörper.

Die Mengen an Anti-HBs sind nach der regulären Impfung mit 3 oder mehr Dosen, aber auch nach überstandener HBV-Infektion meistens eher gering. Für die Erprobung der Impfstoffe und die HBV-Diagnostik wurden hochempfindliche, quantitative Anti-HBs-Tests benötigt. Da zunächst die Ergebnisse zwischen den Labors stark schwankten, wurde ein 1. Internationaler Standard (IS) mit 100 willkürlich definierten International Units (IU) pro mL eingeführt. Er bestand aus einem hochtitrigen HBIG, welches eine erwiesene Schutzwirkung in der passiven Immunisierung von HBV-exponierten Empfängern hatte. Die Spender hierfür waren damals durch eine HBV-Infektion und eventuell auch durch unabsichtliche Injektion von HBsAg-haltigen Blutprodukten ungewollt immunisiert worden. In der diagnostischen Praxis erwies sich eine IU/mL als sehr hohe Anti-HBs-Konzentration, die bei vielen Geimpften nicht erreicht wurde. Die Immune Assays konnten dagegen zuverlässig 0,01 IU/mL messen, einige sogar bis herab zu 0,0001 IU/mL, z. B. der von Abbott. Daher wird die Konzentration meist als mIU/mL oder IU/L angegeben. Eine scharfe Grenze der Anti-HBs-Konzentration, ab welcher ein weitgehender Schutz vor HBV-Infektion erzielt worden war, sei es durch HBIG oder aktive Impfung, konnte nicht ermittelt werden, aber als internationaler Konsens wird ein Wert von 10 mIU/mL als Zeichen einer HBV-Immunität erachtet. Leider folgern die meisten Diagnostiker daraus, dass Werte < 10 mIU/ml Anti-HBs negativ sind. Niedrigere Werte sind aber durchaus von Belang, zumindest als Merkmal eines boosterfähigen Immungedächtnisses.

Umgekehrt sind Durchbrüche bei etwas höhen Werten nicht ganz selten, je nach Immunkonstitution der Probanden, z. B. bei Hämodialysepatienten. Daher empfehlen einige nationale Leitlinien, so auch die deutsche [49], eine Boosterimpfung bei Personen mit hohem Infektionsrisiko bei Werten unter 100 IU/L. Was eine IU Anti-HBs hinsichtlich der HBV-*neutralisierenden* Schutzwirkung bedeutet, ist nicht bekannt. Die Göttinger Gruppe hat gemessen, dass eine IU des 1. IS 0,906 µg HBsAg *binden* kann; wesentliche Unterschiede zwischen den HBsAg-Subtypen gab es damals nicht [64]. Dieses Ergebnis kann helfen, z. B. grob abzuschätzen, mit wie viel HBsAg-haltigem Blut, z. B. durch eine Fehltransfusion, ein Patient belastet werden kann, ohne infiziert zu werden. Eine klare Beziehung zwischen den IU und derjenigen Anti-HBs-Dosis, die eine bekannte Zahl von infektiösen HBV-Partikeln zu 50 % in einer hoch suszeptiblen Zelllinie neutralisieren kann, ist aber nie ermittelt worden, nicht zuletzt deswegen, weil es bis vor Kurzem solche Zelllinien nicht gab.

Der 1. IS ist seit Langem aufgebraucht und wurde durch den 2. IS ersetzt. Auch dieser IS ist ein HBIG, aber diesmal wurde es aus geimpften Spendern gewonnen. Man kann bezweifeln, dass der 2. IS Antikörper gegen die PräS-Epitope hat und dass er Antikörper gegen die HBsAg-Subtypdeterminanten *y* oder *r* oder gar *a4* hat. Die IU bleibt weiter willkürlich definiert, ist nunmehr aber vielleicht in unklarer Weise verändert. Zumindest erneute Bindungsstudien, Subtypanalysen und nach Möglichkeit Neutralisationstests wären wünschenswert.

## Perspektiven für bessere HB-Impfstoffe

### Erneute Erprobung des präS1-haltigen Impfstoffs aus Israel.

Die Produktion des Bio-HepB3-Impfstoffs wurde seit 1993 stetig weitergeführt und neuerdings von VBI Vaccines unter der Bezeichnung Sci-B-Vac® übernommen. In einem großen multizentrischen Feldversuch mit 1607 Teilnehmern wurde erneut die Überlegenheit von Sci-B-Vac® gegenüber Engerix-B®, speziell bei älteren Probanden belegt [35]. Vielleicht noch wichtiger für die Eradikation des HBV ist eine kleine Studie aus Israel an Neugeborenen von HBV-infizierten Müttern [36]. Nach Impfung mit Sci-B-Vac® und HBIG waren nur ein von 89 Kindern nach einem Jahr HBsAg-positiv, dagegen 5 von 82 nach Engerix-B® plus HBIG (*p* = 0,05). Wegen der geringen Zahl von Müttern mit hoher Virämie bei der Geburt konnte kein statistisch signifikanteres Ergebnis erhalten werden. Dennoch könnte diese Studie ein Anlass für weitere Erprobungen in einer Kohorte mit zahlreicheren hochvirämischen Müttern sein.

### Alternative PräS1-Impfstoffe.

Hier sind 2 Konzepte zu nennen, die noch nicht die klinische Erprobung erreicht haben, aber zumindest vielversprechend sind. Die Gruppe von Rudolf Valenta in Wien beschäftigt sich mit Impfstoffen gegen Allergene und kam auf die Idee, die PräS-Domänen wegen ihrer gut charakterisierten T‑Zellepitope [63] als Trägerprotein für die zu bekämpfenden Allergene, z. B. Graspollen, zu verwenden. Ein Vorteil der PräS-Domänen ist ihre unkomplizierte Expression in Bakterien. Als erfreulichen Nebeneffekt der Immunisierung von allergischen Probanden mit ihrem experimentellen Impfstoff *BM32*® fand die Gruppe im Jahr 2016 HBV-neutralisierende Anti-PräS1-Antikörper [38]. Bei weiteren Probanden wurde gezeigt, dass diese Antikörper tatsächlich gegen die Rezeptorbindungsstelle im PräS1 gerichtet sind und mit allen HBV-Genotypen von A bis H reagieren [39]. Da es schon erste Erprobungen im Menschen mit Graspollenallergie gibt, sollten nunmehr spezielle Studien zur Schutzwirkung gegen HBV-Exposition möglich sein. Die Bestimmung des Anti-HBs gibt hier naturgemäß keine Informationen. Eine gewisse Schwäche der PräS1-Domäne ist ihre relativ geringe B‑Zellantigenität und -immunogenität. Vielleicht ist die Graspollenkomponente in BM32® ein wirksamer B‑Zellepitopträger.

Noch geeigneter ist in dieser Hinsicht wohl das Core-Partikel des HBV (HBcAg). Chronisch infizierte HBV-Träger produzieren meistens riesige Anti-HBc-Mengen, die wegen der HBs-Hülle des HBV nicht schützen, aber – soweit bekannt – auch nicht schaden. HBcAg-Partikel stimulieren die Antikörperbildung auch ohne T‑Zellepitope, enthalten aber auch zusätzlich T‑Zellepitope. HBcAg-Partikel exponieren ihr einziges B‑Zellhauptepitop an der Spitze von „Spikes“. Es kann gentechnisch oder biochemisch durch viele andere Peptidsequenzen ersetzt oder besetzt werden. Dieter Glebes Gruppe in Gießen setzte in Zusammenarbeit mit der Gruppe von Paul Pumpens in Riga Teilpeptide aus der PräS1-Domäne in die Spitze dieser Spikes ein, immunisierte Mäuse mit diesem rekombinanten HBcAg und maß die neutralisierende Wirkung der erhaltenen Antiseren. Auf diese Weise konnten die Epitope der neutralisierenden PräS1-Antikörper in der HBV-Rezeptorbindungsstelle exakt kartiert werden. Die in den immunisierten Mäusen erhaltenen wirksamsten Antiseren konnten in einer Verdünnung von 1:5000 die Infektiosität von 10^6^ HBV-Partikeln für suszeptible Leberzellkulturen neutralisieren, was einem „normalen“ Antiserum oder HBIG mit dem enormen Titer von 100.000 IU/L Anti-HBs entspräche [65].

Das Konzept wurde von David Milichs Gruppe mit dem Ziel eines therapeutischen HBV-Impfstoffs vertieft [66]. Die Gruppe verwendete die ähnlich strukturierten Core-Partikel des Woodchuck-Hepatitis-Virus (WHV) als Träger für die neutralisierenden HBV-PräS1-Epitope und behandelte damit ein immuntolerantes HBV-transgenes Mausmodell für die menschliche chronische Hepatitis B. Die Tiere entwickelten neutralisierende Antikörper, die chimäre Mäuse mit menschlichen Hepatozyten vor HBV schützten.

Diese beiden PräS1-Impfstoffkonzepte ohne S‑HBsAg zielen auf eine Immuntherapie der chronischen Hepatitis B (CHB) und sind zurzeit wohl noch weit von einer klinischen Testphase entfernt. Es spräche jedoch theoretisch nichts dagegen, diese Impfstoffe bei erwiesener therapeutischer Wirksamkeit auch für die Prophylaxe, speziell postexpositionell bei den Neugeborenen von hochvirämischen Müttern zu entwickeln.

### Wirksamere Adjuvanzien.

Mittlerweile ist in den USA und Europa der Impfstoff Heplisav-B® von Dynavax verfügbar, der konventionelles S‑HBsAg P24 aus Hefe enthält, der aber statt Aluminiumverbindungen ein CpG-Oligonucleotid (einen Toll-like-Rezeptor-(TLR-)9-Agonisten) als Adjuvans enthält, das die Immunogenität so kräftig steigert, dass im Normalfall nur 2 Dosen nötig sind. Trotzdem ist die Anti-HBs-Produktion in den Geimpften besser als mit 3 Dosen von Engerix-B®, auch bei Personengruppen, die eine schwächere Anti-HBs-Antwort zeigen, wie z. B. Patienten mit chronischen Leberkrankheiten [33]. Bei einem besonders schwierig zu immunisierendem Kollektiv, wie z. B. Hämodialysepatienten, wurden 4 Dosen gegeben und damit eine hohe Serokonversionsrate von 89 % sowie ein Anti-HBs-Titer von ca. 1000 IU/L erreicht [34]. Neben CpG wurde bei dieser Patientengruppe von GSK auch AS04 (3-O-desacyl-4′-monophosphoryl Lipid A (AS04), ein TLR-4-Agonist) als Adjuvans in 4 Dosen des Impfstoffs Fendrix® verwendet [32].

Daneben wurden von GSK ähnliche, noch stärker wirkende Adjuvanzien (AS01, AS03) zusammen mit dem P24-HBsAg erprobt. Damit genügten ähnlich wie bei Heplisav-B® 2 Dosen, um 100 % Serokonversion und 100-mal höhere Anti-HBs-Titer als mit dem Aluminiumadjuvans zu erzielen. Hier sind allerdings verstärkte Nebenwirkungen zu verzeichnen [67]. Es bleibt offen, ob bessere HBV-Antigene, wie z. B. bei Sci-B-Vac®, zusammen mit stärkeren Adjuvanzien noch bessere Ergebnisse bringen könnten.

### Neutralisierende humane monoklonale Antikörper.

HBIG von HB-geimpften Spendern ist nicht ausoptimiert, sodass eine wesentliche Verbesserung der passiven Immunisierung möglich erscheint. Aus dem Lymphozyten von HBV-Rekonvaleszenten wurden neuerdings humane monoklonale Anti-PräS1-Antikörper isoliert, die infektiöses HBV in einem Zellkultursystem neutralisieren konnten [68]. Noch breiter schützende monoklonale neutralisierende Anti-HBs-Antikörper konnten aus HBV-Trägern (Natural Controllers) isoliert werden, die ohne Therapie nach einer Phase chronischer HBs-Antigenämie das HBsAg verloren und Anti-HBs gebildet haben. Interessanterweise waren monoklonale Anti-HBs-Antikörper von HB-Geimpften weniger wirksam neutralisierend. Die Befunde belegen, dass die Möglichkeiten der passiven Immunisierung bei Weitem noch nicht ausgeschöpft sind [69].

***HBV-Antigene exprimierende DNA oder Virusvektoren als HBV-Impfstoffe*** sind seit Langem in Tiermodellen vielfach untersucht worden. Die Forschung am Menschen war bislang noch nicht soweit gediehen, dass sie hier diskutiert werden sollten. Das Beispiel Coronaviren zeigt jedoch, dass sich das rasch ändern könnte.

## Fazit

Nach schwierigen Anfängen gelang es vor 50 Jahren mithilfe der damals neu entwickelten Molekularbiologie, schutzrelevante HBV-Antigene zu identifizieren und hochwirksame Impfstoffe gegen HBV zu entwickeln. Trotz der weltweit großen Erfolge der aktiven und passiven Immunisierung gibt es noch erhebliche Schwächen in der Immunisierung gegen HBV. Daher sollte die Entwicklung noch wirksamerer HBV-Antigene und entsprechender Antikörper im Verbund mit aktiven T‑Zellepitopen verstärkt werden. Die heute vorwiegend verwendeten Präparate beruhen auf dem Kenntnisstand von vor 40 Jahren und sind erheblich verbesserungsfähig. Die Anwendung der neuesten Konzepte mit mRNA- oder Vektorimpfstoffen wie bei COVID-19 steht noch aus.

### Infobox 1 Meilensteine in der Entwicklung der Hepatitis-B-Impfung. Erläuterungen siehe Text

Klinik und Epidemiologie der Hepatitis B.

- 1885: 2 durch Lürmann und Jehn klar beschriebene Ausbrüche von Gelbsucht nach Pockenimpfung in Deutschland, mutmaßlich durch infektiöses Humanserum
- 1900–1942: zahlreiche Ausbrüche nach Injektionen mit serumverunreinigten Materialien
- 1944–1970: experimentelle Infektion von „Freiwilligen“ mit Hepatitiserregern, Unterscheidung von 2 Formen der Hepatitis: infektiöse Hepatitis und Serumhepatitis
- 1947: offizielle Benennung der beiden Formen als Hepatitis A und B

1. Generation der Hepatitis-B-Impfstoffe aus Plasma.

- 1965: Entdeckung des „Australia-Antigens“ (AuAg, heutige Bezeichnung HBsAg) durch Harvey Alter und Baruch Blumberg in einem Aborigine
- 1968: Entdeckung des Serumhepatitisantigens bei Patienten mit Posttransfusionshepatitis durch Alfred Prince; Übereinstimmung des Antigens mit dem „Australia-Antigen“
- 1971: experimentelle Infektion von geistig behinderten Kindern mit HBV durch Saul Krugman, daneben auch partielle Immunisierung gegen HBV mit erhitztem australia-antigen-haltigem Serum
- 1972–1976: Verwendung von Schimpansen für die Messung des HBV-Infektionstiters (ID 50) und für die experimentelle Immunisierung mit gereinigtem HBsAg durch Robert Purcell, John Gerin am NIH und durch Maurice Hilleman bei MSD
- 1976–1980: 2 sehr erfolgreiche Schutzversuche mit gereinigtem HBsAg aus HBV-Trägerplasma als Impfstoff in HBV-gefährdeten Kollektiven durch Philippe Maupas und durch Wolf Szmuness

2. Generation: gentechnische Impfstoffe mit dem kleinen HBsAg-P24-Protein.

- 1970: Entdeckung virusartiger 45 nm-Partikel in AuAg-haltigen Seren durch David Dane
- 1973: Entdeckung der endogenen DNA-Polymerase und einer kleinen ringförmigen DNA in den Dane-Partikeln durch William Robinson
- 1978: Klonierung und Sequenzierung der HBV-DNA durch die Gruppen von Pierre Tiollais, Kenneth Murray und William Rutter, Identifizierung des S‑Gens für das kleine („small“) Haupthüllprotein des HBsAg P24
- 1982–1984: Herstellung des HBsAg P24 (SHBs) in Hefezellen, die das S‑Gen exprimieren, bei den Firmen MSD und GSK. Nachweis der Schutzwirkung eines Impfstoffs mit diesem „rekombinanten“ HBsAg

3. Generation: gentechnische Impfstoffe mit allen 3 HBsAg-Proteinen.

- 1984: Nachweis des großen HBsAg-Proteins (P39/GP42, LHBs) mit der PräS1-Domäne als wesentliche Komponente der Dane-Partikel durch Klaus Heermann und Wolfram Gerlich
- 2003: Nachweis der neutralisierenden Wirkung von Anti-PräS1-Antikörpern auf die HBV-Infektiosität in Leberzellkulturen durch Dieter Glebe
- Ab 1994: Gemeinsame Expression aller 3 HBs-Proteine in Säugerzellen als nichtinfektiöse subvirale Partikel und Erprobung als Impfstoff in Versuchstieren
- 1997–2021: Erprobung von Impfstoffen der 3. Generation mit verbesserter Immunogenität und Schutzwirkung in verschiedenen Probandengruppen
